# Intravitreal injections during COVID-19 outbreak: Protective measures, total duration of care and perceived quality of care in a tertiary retina center

**DOI:** 10.1177/11206721211003488

**Published:** 2021-03-22

**Authors:** Paul Denys, Alexandra Miere, Donato Colantuono, Camille Jung, Eric H. Souied

**Affiliations:** 1 Department of Ophthalmology, Centre Hospitalier Intercommunal de Créteil, University Paris Est Créteil, Créteil, France; 2 Clinical Research Center, GRC Macula, and Biological Resources Center, Centre Hospitalier Intercommunal de Créteil, Créteil, France

**Keywords:** Intravitreal injections, age-related macular degeneration, diabetic macular edema, treatment burden, patient satisfaction, COVID-19

## Abstract

**Purpose:**

To assess patient satisfaction regarding the sudden reorganization of care during the COVID-19 pandemic in the outpatient intravitreal injection (IVI) clinic.

**Methods:**

A survey of patients with ongoing IVIs for retinal diseases was carried out between April 23rd and May 12th, 2020. We designed a questionnaire to assess patient satisfaction concerning: personal protective equipment (PPE), social distancing, the perceived quality of care, and the total time spent in the department, using a Likert scale. We also collected the time spent per patients in the outpatient IVI clinic.

**Results:**

A hundred and twenty-seven eyes of 108 patients were included. The mean time spent in the IVI outpatient clinic was 31.87 +/− 16.61 min. In our survey, 99.1% of the patients were satisfied (highly satisfied or satisfied) with the new type of care provided, 89.8% with the duration of care, and 93.5% with the PPE. Satisfaction was associated with total time spent in hospital (*p* = 0.005), with dissatisfied patients spending about 50% more time in the hospital than satisfied patients (43.91 min vs 30.50 min).

**Conclusion:**

Despite the crisis-related adjustment, our survey revealed high patient satisfaction with PPE, quality of care, and total time spent in outpatient IVI clinic.

## Introduction

Since December 2019, a severe acute respiratory syndrome due to a novel, highly contagious coronavirus (SARS-CoV-2) has led to a major health crisis throughout the world.^1^ To face this issue the French government has decided to place the whole population under quarantine.

SARS-CoV-2 is transmitted through large droplets, contacts, and fomites.^2^

Among healthcare workers, ophthalmologists are particularly at risk of infection and transmission of the virus due to close contact with patients during the examination.

Our department is a tertiary referral center for the diagnosis and treatment of retinal disease. Elderly population and adults with various comorbidities, such as obesity, arterial hypertension, or diabetes mellitus appear to be at high risk of severe COVID-19 infection and mortality.^3^ Even so, added to the risk related to the vital prognosis during the COVID-19 outbreak in these patients, the visual prognosis is also at risk for both patients with neovascular age-related macular degeneration (AMD) and any other retinal vascular disease.

The introduction of anti-VEGF therapies in the past decade shifted the prognosis of many retinal diseases such as AMD, retinal vein occlusion, and diabetic macular edema. Many treatment regimens have been proposed to modulate the rhythm of intravitreal injection (IVI).

On one hand, the Treat-and-Extend dosing regimen consists of an initial monthly loading dose of at least three IVI followed by variable treatment intervals, according to the evolution of the disease. On the other hand, the Pro re nata (PRN) regimen, consists of monthly monitoring and injection if exudative signs or macular edema are present.

Given a large number of daily consultations, IVI, and the inherent proximity between high-risk patients, the outpatient clinic activity was carefully reorganized, to ensure protection for physicians and patients, according to the recommendations of the national and international Ophthalmology societies.^4,5^ Each patient was summoned every 15 min and asked to be present at the exact time of the appointment. The waiting room was set up so that there was a minimum distance of 2 meters between each patient. Accompanying persons were asked to keep outside the outpatient clinic. All these measures were taken to guarantee social distancing measures and to limit the number of patients in the department at the same time. The question was to know if this new organization of patient flow was acceptable or not for our patients.

Our study aimed to assess the satisfaction of patients in the outpatient IVI clinic in terms of duration of care, personal protective equipment, and social distancing measures, global quality of care during the COVID-19 pandemic.

## Methods

The survey has been conducted between April 23rd and May 12th, 2020 in the outpatient intravitreal injection clinic in the Department of Ophthalmology at Créteil Hospital, Créteil, France, by randomly selecting patients from the IVI clinic. All patients had been previously instructed to come at the precise hour of the scheduled IVI and by themselves.

Inclusion criteria were: ongoing treatment during the COVID-19 outbreak with IVI for neovascular AMD, retinal vascular disease, pachychoroid spectrum disease, and other retinal diseases. There were no exclusion criteria except the refusal to participate.

We collected information on patient demographics (birth sex, age), ophthalmic diagnoses (current age, follow up period, number of intravitreal injections in the past year, type, and subtype of retinal disease). We also collected the total time spent by patients in the outpatient IVI clinic and we checked if their arrival time coincided with the actual appointment time. All responses were anonymous and the database of results is available upon request from the corresponding author.

As a reference group, we compared our data during the COVID-19 crisis to data from a prospective, observational study conducted between the October 12th and October 16th, 2017 in the IVI outpatient clinic to investigate the duration of care. The survey consisted of 43 patients. This study was used as a reference for the waiting time in the IVI outpatient clinic under normal conditions.

Patient satisfaction was measured by asking three questions regarding time spent in the outpatient IVI clinic, SARS-CoV-2 preventive measures (face masks, hydro-alcoholic gel, distancing measures, etc.) and overall satisfaction with the quality of care during COVID-19 pandemic. Responses were collected using the 5-point Likert scale: 5 (Very satisfied), 4 (Satisfied), 3 (Neutral), 2 (Dissatisfied), or 1 (Completely dissatisfied). Given that certain patients came in regularly to our department for chronic retinal disease, we compared the number of IVIs per patient over the past year, the total follow up period, as well as the monophtalmic status of the patient to the level of satisfaction.

### Statistical analysis

Data from the paper questionnaires were double entered in a Microsoft Excel spreadsheet.

We used descriptive analysis to describe patient characteristics and item scores. Mann-Whitney and Chi^2^ tests were conducted to compare the differences between clinical characteristics and satisfaction/dissatisfaction. For this purpose, we distinguished two groups: group one consisted of highly satisfied and satisfied patients, and group 2 consisted of dissatisfied or very dissatisfied patients. Statistical analysis was performed using STATA version 13.0 (Texas, US). *P* < 0.05 was retained as significant.

## Results

A hundred and twenty-six eyes of 108 patients were included on 364 IVI. The mean age was 74.26 +/− 10.67 (range 40–93, 45 males, 63 females). Included eyes had a diagnosis of neovascular AMD in 66 eyes: 41 type 1 macular neovascularization (MNV), 14 type 2 MNV, and nine type 3 neovascularization, one mixed type 1 and 2 MNV and one hematoma. Macular edema (ME) secondary to retinal vascular disease was present in 34 eyes: 20 eyes with diabetic macular edema (DME) and 14 eyes with ME secondary to retinal vein occlusion (RVO) (eight branch RVO, six central RVO). Pachychoroid disease spectrum was present in 14 eyes: six polypoidal choroidal vasculopathy/ aneurysmal type 1 neovascularization (PCV/AT1), two central serous chorioretinopathy, and six pachychoroid neovasculopathy. Finally, less common diagnoses have also been included such as six eyes with neovascularization secondary to pathologic myopia and to one osteoma, one macroaneurysm, three eyes with neovascularized pattern dystrophy, one eye with neovascularization secondary to angioid streaks. These results are summarized in Table 1. Of all included patients 11.1% were monophthalmic and 15.7% had been followed for less than a year, while 37% had a follow up of 1 to 5 years, 38% for 5 to 10 years, and 10.2% for more than 10 years.

**Table 1.** Patients demographics and clinical characteristics.

Clinical features	Value
Mean age	74.26 (+/−10.67)
Retinal disease	
AMD	66 (52.38%)
MNV1	41 (32.54%)
MNV2	14 (11.11%)
MNV3	9 (7.14%)
Mixed type 1 and 2	1 (0.79%)
Hematoma	1 (0.79%)
Retinal vascular disease	34 (26.98%)
Diabetes	20 (15.87%)
Retinal vein occlusion	14 (11.11%)
Pachychoroid	14 (11.11%)
VPC	6 (4.76%)
CSC	2 (1.59%)
Pachychoroid neovasculopathy	6 (4.76%)
Others	
Neovascularized myopia	12 (9.52%)
Macroaneurysm	6 (4.76%)
Osteoma	1 (0.79%)
Neovascularized pattern dystrophy	1 (0.79%)
Neovascularized angioid streaks	3 (2.38%)
	1 (0.79%)
Sex	
Female	58.33%
Male	42.69%
Follow up period	
<1 year	15.74%
1 to 5 years	37.04%
5 to 10 years	37.96%
>10 years	10.19%
Mean number of IVI in the last year	6.80 (+/−2.30)
Number of monophtalmic patients	13 (11.11%)
Number of accompanied patients	35 (32.41%)

The mean time spent in the IVI outpatient clinic was 31.87 +/− 16.61 min. Patients arrived an average of 5.3 +/− 19.8 min earlier than their appointments and 31.2% of patients were accompanied.

In October 2017 the mean time spent in the IVI outpatient clinic was 108.25 +/− 36.46 min. Waiting time in the department during the COVID-19 outbreak was statistically inferior (*p* < 0.001, Mann Whitney test) to the waiting time in 2017.

Concerning personal protective equipment (PPE), 66 patients (61.11%) were highly satisfied, 35 patients were satisfied (32.41%), and seven patients were dissatisfied (6.48%).

Regarding total time spent in the IVI outpatient clinic, 70 patients (64.81%) reported high satisfaction concerning the total time spent in the outpatient clinic, 27 (25%) patients were satisfied while 10 patients (9.26%) were dissatisfied and one patient (0.93%) was very dissatisfied with waiting time.

Concerning the perceived quality of care despite the Covid-19 outbreak, 73 patients (67.59%) reported being highly satisfied, 34 patients (31.48%) were satisfied and one patient (0.93%) was dissatisfied. These outcomes are resumed in Table 2.

**Table 2.** Patient response to the questionnaire using Likert scale.

	Very satisfied	Satisfied	Neutral	Dissatisfied	Very dissatisfied
Q1*	66 (61.11%)	35 (32.41%)	0	7 (6.48%)	0
Q2^†^	70 (64.81%)	27 (25%)	0	10 (9.26%)	1 (0.93%)
Q3^‡^	73 (67.59%)	34 (31.48%)	0	1 (0.93%)	0

* Q1: patient satisfaction with SARS-CoV-2 preventive measures (face masks, hydro-alcoholic gel, distancing measures, etc.)

† Q2: patient satisfaction regarding time spent in the outpatient IVI clinic.

‡ Q3: overall satisfaction with the quality of care during Covid-19 pandemic.

Waiting time in the IVI outpatient clinic was associated in a statistically significant manner with patient satisfaction/dissatisfaction (*p* = 0.005, Mann Whitney test) concerning their total time spent in the IVI outpatient clinic. Satisfied patients spent an average of 30.50 min in the IVI outpatient clinic while the dissatisfied group spent an average of 43.91 min in the IVI outpatient clinic.

There was no statistically significant difference in terms of IVI number within the last year, total follow up period and satisfaction/dissatisfaction status with PPE, time spent in the hospital, and perceived quality of care during COVID-19 pandemic.

We also thought that monophthalmic patients were more anxious about their visual outcome and this might influence their answers. However, there was no correlation between monophtalmic status and patient satisfaction. These results are summarized in Tables 3 and 4.

**Table 3.** Evaluation of the correlation between patient satisfaction with the time spent in hospital and the IVI number within the last year using a Mann-Whitney test.

	Q1*		Q2^†^		Q3^‡^	
	*Z*	*p*	*Z*	*p*	*Z*	*p*
Total time spent in the IVI outpatient clinic	−0.12	0.9056	2.790	0.0053	0.369	0.71
IVI number within the last year	0.45	0.6512	−0.12	0.90	1.37	0.17

* Q1: patient satisfaction with SARS-CoV-2 preventive measures (face masks, hydro-alcoholic gel, distancing measures, etc.)

† Q2: patient satisfaction regarding time spent in the outpatient IVI clinic.

‡ Q3: overall satisfaction with the quality of care during Covid-19 pandemic.

**Table 4.** Evaluation of the correlation between patient satisfaction with the follow up period and the monophtalmic status using Chi^2^ test.

	Q1*		Q2^†^		Q3^‡^	
	*X* ^2 statistic value^	*p*	*X* ^2 statistic value^	*p*	*X* ^2 statistic value^	*p*
Follow up period	4.48	0.21	0.064	0.99	1.72	0.63
Monophtalmic status	1.02	0.31	0.10	0.75	0.14	0.71

* Q1: patient satisfaction with SARS-CoV-2 preventive measures (face masks, hydro-alcoholic gel, distancing measures, etc.).

† Q2: patient satisfaction regarding time spent in the outpatient IVI clinic.

‡ Q3: overall satisfaction with the quality of care during Covid-19 pandemic.

Between April 23rd and May 12th, 2020, we performed 364 IVIs in our tertiary center. During the same period in 2019, we performed 560 IVIs. We thus note a 35% decrease in the number of IVIs between 2020 and 2019 over the given period.

## Discussion

In this cross-sectional study, we reported satisfaction in patients undergoing intravitreal injections during the COVID-19 pandemic, using a questionnaire, in regards to PPE, the total duration of care, and perceived quality of care. While previous studies have focused on the psychological impact (fear, anxiety) of COVID-19 outbreak on healthcare workers,^6,7^ our study focused on these patients’ perception of care during the current health crisis.

The sudden spread of SARS-CoV-2 around the world, and especially in western Europe, and the subsequent lockdown, led us to rethink the organization of the Ophthalmology Department. Among the global 216.6 million (80% uncertainly interval 98.5 to 359.1 million) population with severe or moderate visual impairment in 2015: 8.4 million (0.9 to 29.5 million) and 2.6 million (0.2 to 9.9 million) had a diabetic retinopathy.^8^ Therefore, AMD and diabetes are two leading causes of blindness, and maintaining continuity of care despite the health crisis and contamination risks remains a priority to limit the impact of the outbreak on patients’ functional and anatomical outcomes.

To provide care with maximum safety for both patients and physicians, several measures have been undertaken. Indeed, two major aspects of reorganization due to the outbreak focused on PPE and social distancing measures and on reducing the total time spent in the hospital, to reduce the risk of contamination. Patients were summoned every 15 min (instead of the usual 7 min), patients were asked to come alone if possible and the waiting room was rearranged to respect social distancing measures Patients were compliant with the indications given before the IVI visit, arriving an average of 5.3 +/− 19.8 min earlier than their appointment and with only 31.2% of patients being accompanied by a family member. The current study investigated patient satisfaction regarding three aspects: PPE and social distancing measures, the total duration of care, and perceived quality of care. Patient satisfaction was not associated with the follow-up period, the number of IVIs within the last year, or their monophtalmic status.

Interestingly, most of the patients were highly satisfied or satisfied with the provided care despite the situation (99.1%), with the total time spent in hospital (89.8%) and with the PPE to prevent from human-to-human contamination by the virus (93.5%). In our series, the mean time spent per patient in the IVI clinic was of 31.87 +/− 16.61 min. The duration of care during COVID-19 outbreak was significantly lower than the baseline in 2017 (*p* < 0.001, Mann Whitney test). We thus noted a reduction of 61.66% of the mean duration of care per patient compared to 2017. Efforts can still be made to further reduce the waiting time, although one of the physicians has managed to achieve a significantly lower waiting time for the patients, of 16 min per patient in average (*p* < 0.001, Mann-Whitney test). The COVID-19 outbreak has therefore forced us to rethink the organization of the IVI outpatient clinic in the most efficient way possible and should serve as a reference for the future.

It is noteworthy to mention that patient satisfaction was indeed associated with the total time spent in the hospital (*p* = 0.005), with dissatisfied patients spending approximately 50% more time in the hospital than satisfied patients (43.91 min vs 30.50 min). These preventive measures to minimize the risk of contagion explain why patients were highly satisfied (67.59%) by the overall perceived quality of care in the IVI outpatient clinic. Although data are insufficient to precisely define the duration of time that constitutes a prolonged exposure, current recommendations suggest that brief interactions are less likely to result in transmission. Therefore, an even lower waiting time would be desirable for patients, as achieved by certain physicians, by constantly inspecting the waiting room.^9^

In conclusion, the current pandemic is one of the greatest challenges that health care workers have ever experienced. The majority of health institutions have had to reorganize their activity and many ophthalmologists have been requisitioned from the medical services to help the general effort in fighting the outbreak. Nevertheless, in retinal diseases, a discontinuity in treatment may lead to a severe loss in visual acuity and sight-threatening complications. Therefore, we have maintained more than 50% of the IVIs, while guaranteeing maximum safety for caregivers and patients.
